# General Anesthesia Obtained by Spinal Application

**Published:** 1918-12

**Authors:** 


**General Anesthesia Obtained by Spinal Application.** By Dr. SAVARIAUD. *Bulletin de la Société de Chirurgie de Paris,* May 7, 1918.

The author insists that great care must be taken in administering anesthesia, so as to avoid post-operative complications. He proceeds as follows : a mask of special cloth is closely fixed upon the face by an elastic band, with a small hole for the passage of the air. In this mask he introduces a compress soaked with ethylchloride 5 cc. and chloroform 1 cc. Unconsciousness is obtained after a few seconds, with no period of excitement. By adding from time to time 1/2 cc. of chloroform, unconsciousness is maintained as long as necessary. Vomiting has proved extremely rare. Never has the author observed post-operative shock or subicterus.

The author, however, still prefers spinal injections whenever these are possible. He uses a 2 per cent. solution containing 1/3 of cocaine, 2/3 of stovaine. He makes the puncture rather low, takes out 12 to 20 cc. of spinal fluid, and injects 1 1/2 to 2 cc. of the solution.

Results are perfect for operations on the inferior limbs and abdomen. Without increasing the quantities of anesthetics, simply by inclining the patients, Dr. Savariaud has obtained some cases of general anesthesia. He thinks that in the future this method may be generalized.

